# Primary Productivity and Precipitation-Use Efficiency in Temperate Grassland in the Loess Plateau of China

**DOI:** 10.1371/journal.pone.0135490

**Published:** 2015-08-21

**Authors:** Xiaoxu Jia, Baoni Xie, Ming’an Shao, Chunlei Zhao

**Affiliations:** 1 Key Laboratory of Ecosystem Network Observation and Modeling, Institute of Geographic Sciences and Natural Resources Research, Chinese Academy of Sciences, Beijing 100101, China; 2 College of Natural Resources and Environment, Northwest A&F University, Yangling 712100, China; 3 State Key Laboratory of Soil Erosion and Dryland Farming on the Loess Plateau, Northwest A&F University, Yangling 712100, China; Institute of Tibetan Plateau Research, CHINA

## Abstract

Clarifying spatial variations in aboveground net primary productivity (ANPP) and precipitation-use efficiency (PUE) of grasslands is critical for effective prediction of the response of terrestrial ecosystem carbon and water cycle to future climate change. Though the combination use of remote sensing products and *in situ* ANPP measurements, we quantified the effects of climatic [mean annual precipitation (MAP) and precipitation seasonal distribution (PSD)], biotic [leaf area index (LAI)] and abiotic [slope gradient, aspect, soil water storage (SWS) and other soil physical properties] factors on the spatial variations in ANPP and PUE across different grassland types (i.e., meadow steppe, typical steppe and desert steppe) in the Loess Plateau. Based on the study, ANPP increased exponentially with MAP for the entire temperate grassland; suggesting that PUE increased with increasing MAP. Also PSD had a significant effect on ANPP and PUE; where more even PSD favored higher ANPP and PUE. Then MAP, more than PSD, explained spatial variations in typical steppe and desert steppe. However, PSD was the dominant driving factor of spatial variations in ANPP of meadow steppe. This suggested that in terms of spatial variations in ANPP of meadow steppe, change in PSD due to climate change was more important than that in total annual precipitation. LAI explained 78% of spatial PUE in the entire Loess Plateau temperate grassland. As such, LAI was the primary driving factor of spatial variations in PUE. Although the effect of SWS on ANPP and PUE was significant, it was nonetheless less than that of precipitation and vegetation. We therefore concluded that changes in vegetation structure and consequently in LAI and/or altered pattern of seasonal distribution of rainfall due to global climate change could significantly influence ecosystem carbon and water cycle in temperate grasslands.

## Introduction

Precipitation is the key factor in controlling primary production of most of the world’s grassland ecosystems, especially in arid and semiarid regions [1,2,3]. It is predicted that global climate change will lead to more frequent extreme precipitation and drought events, with significant effects on ecosystem processes [4,5]. Aboveground net primary productivity (ANPP), a key integrative measure of ecosystem function, is reported to be greatly affected by changes in mean annual precipitation (MAP) and/or precipitation seasonal distribution (PSD) [6,7,8,9,10,11]. There is a growing interest to explore relations between both ANPP-MAP and ANPP-PSD under global climate change. While the patterns of MAP-ANPP relations are highly variable, the underlying processes of these relations are not yet fully understood. For example, some studies have reported simple linear relation between MAP and ANPP [12,13,14,15] and others have noted exponential relation [10,16,17] for the same temperate grasslands in China.

The response of grassland ANPP to change in PSD is still controversial. For example, Thomey et al. [18] and Guo et al. [10] noted that more concentrated PSD (i.e., large, infrequent rainfall events) attenuated water stress which, in turn improved Chihuahuan desert and Eurasian temperate grassland ANPP. However, the reverse was observed for Kansas tallgrass prairie where more even PSD favored higher ANPP [3,19]. Although extreme rainfall regimes induce greater dry intervals between rainfall events, semiarid steppes are not particularly sensitive to increase in rainfall event intervals because semiarid steppe plants are well adapted to extended periods of intense water stress [2]. Besides, several studies have shown that concentrated rainfall regimes lead to deep infiltration of precipitation water into the soil layer and less proportional loss to evaporation, thus increasing the amount and duration of water in the soil for subsequent plant uptake [10,18,19,20]. However, all of these studies assumed no dramatic increase in runoff with larger rain events [9]. The conditions in the Loess Plateau are possibly different from those in other regions. The Loess Plateau is highly complex in topography and landform and the loess soil is easily prone to erosion. Also the few larger rainfall events could induce excessive water loss as overland flow. Thus the effects of altered PSD pattern on grassland ANPP in the Loess Plateau could be different from those in other areas. However, the relative effects of MAP and PSD on ANPP variations in the Loess Plateau are still largely unknown.

Precipitation-use efficiency (PUE), the ratio of ANPP to precipitation, is a useful index that explains the relation between ecosystem carbon and water cycle [15,17]. PUE is closely linked to both plant physiological characteristics and physical water loss processes [14,21,22]. In general, PUE tends to decrease spatially with increasing aridity and potential evapotranspiration [23]. However, recent reports have strongly challenged this point [7,13]. For example, Huxman et al. [7] showed decreasing mean PUE from deserts, grasslands to forests with increasing MAP in North and South America. Hu et al. [17] observed that when the range of MAP and ecosystem type narrows toward dry end, the spatial variation in PUE shifts from decreasing to increasing one with increasing MAP. Spatiotemporal variations in PUE are therefore variable due to the controlling physiological and physical processes of PUE which vary with the scale of analysis. While previous studies have mainly focused on the relation between PUE and MAP, little remains known about the response of PUE to altered PSD patterns. Also the lack of knowledge on PUE-PSD relation inhibits the understanding of how climate change affects ecosystem carbon and water cycle.

PUE is linked to many factors, including vegetation composition, edaphic conditions and biogeochemical constraints [7,13,23,24]. For example, communities with higher diversity of plant species and functional groups could have greater PUE due to increased ANPP [25]. Hu et al. [17] showed that vegetation cover significantly affects spatial variations in PUE along 4500 km of grassland transect. Leaf area index (LAI) is a primary factor controlling spatiotemporal variations in ecosystem water use efficiency. Studies in four grassland ecosystems in China have shown that LAI has a regulatory effect on the ratio of transpiration to evapotranspiration [22,26]. Soil properties, such as texture, water-holding capacity and depth are major determinants of soil water availability and could therefore have significant effect on PUE [1,6,23]. Although the effects of above biotic and/or abiotic factors on ANPP and PUE have been widely studied, the relative importance of the factors in the spatiotemporal patterns of ANPP and PUE is not fully understood [15]. PUE patterns and the associated driving factors generally vary from site to site.

The Loess Plateau is ca. 35% comprised of grassland. It is a vital component of China’s grasslands and it is critical for controlling soil and water erosion. Despite its significance, little has been done to address the spatial variations in ANPP and PUE in the grasslands in the Loess Plateau. Precipitation is the only source of water for plant productivity in the arid Loess Plateau region. Plants in the region use only a small fraction of the rainfall because of high evaporation and runoff. Thus this study hypothesized that ANPP and PUE increase with increasing MAP. This is because plants have high growth rates with increasing MAP and subsequently more dense vegetation canopy. Dense canopies induce more precipitation water use in transpiration which in turn results in higher productivity.

Besides, there is generally severe soil and water erosion in the Loess Plateau due to complex landforms, loose soil and sparse vegetation conditions. In the region, there is excessive waste of extreme and concentrated precipitation as overland flow [27,28]. Thus this study also hypothesized that more even PSD will favor higher ANPP and PUE. With few but large rainfall events, more precipitation water is lost to runoff and less used for plants growth. This condition is likely to decrease ANPP and PUE in the Loess Plateau. The main objective of this study was to analyze spatial variations in ANPP and PUE using environmental and ANPP data from different grassland types in the Loess Plateau.

## Materials and Methods

### Ethics statement

The sampling fields belong to “Grain-for-Green” program and permission for plant and soil sampling was obtained by the Institute of Soil and Water Conservation, Chinese Academy of Sciences & Ministry of Water Resources of PRC. The sampling fields in this study did not include endangered or protected species.

### Study area

The Loess Plateau is in Northwestern China and covers latitudes 33°43’–41°16’N and longitudes 100°54’–114°33’E with a total area of 620,000 km^2^, accounting for some 6.5% of the area of China. The region lies in the temperate, arid and semi-arid continental monsoon zones. The mean annual temperature range is 3.6 to 14.3°C and MAP from northwest to southeast is 150–800 mm. About 55–78% of MAP falls in June through September as high intensify rainstorm. The soil is mainly loess, with clay-loam as the most common texture and then sandier soils in the northwest and clayier soils in the southeast. The region has a complex topography, including sub-plateaus, basins, hills and gullies of altitude range of 200–3000 m above mean sea level [29]. Much of the study area experiences severe soil and water erosion, resulting in land degradation and loss of soil fertility. For soil and water erosion control and also for ecosystem restoration, Chinese government initiated an extensive ecological rehabilitation program (the “Grain-for-Green” program), in 1999 in the region. The program has been operating for 15 years now and the natural ecology in most parts of the Plateau is gradually improving.

**Fig 1:**
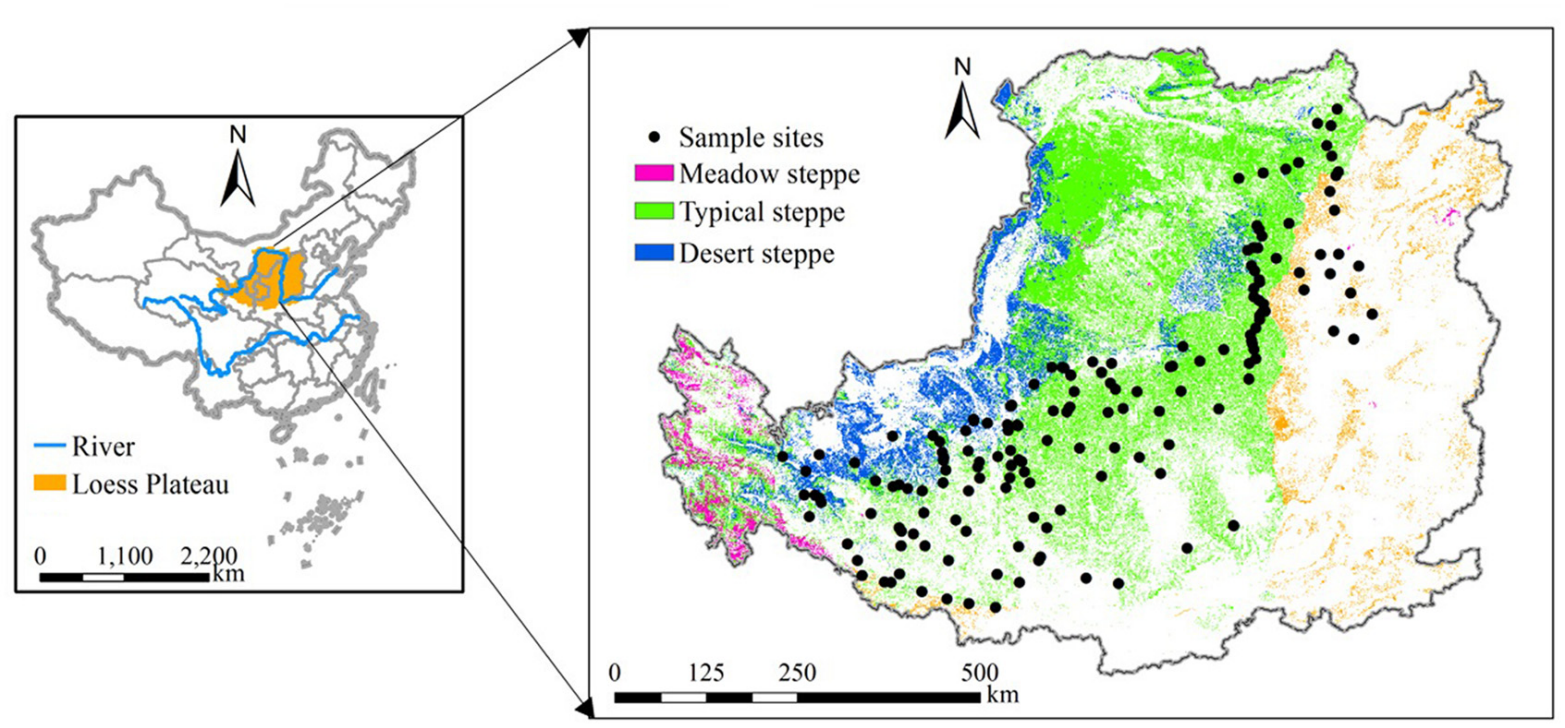
Location of the Loess Plateau in China (left plate) and the distribution of the 188 sampling sites where *in situ* measurements of aboveground net primary productivity were taken in the study area (right plate).

From southeast to northwest, the vegetation changes from forest through forest steppe to typical steppe and then to desert steppe type. Meadow steppe is mainly in the west of the Plateau (Fig 1). The meadow steppe has the highest plant biodiversity, with dominant species of *Festuca ovina*, *Filifoliumsibiricum Kitam*, *Stipa Baicalensis*, *Bothriochloa ischaemum*, *Artemisia giraldii*, *Spodiopogon sibiricus*, and *Leymus secalinus*. The typical steppe has moderate plant biodiversity that is dominated by *Stipa bungeana*, *Artemisia giraldii*, *Thymus mongolicus*, *Lespedeza davurica*, *Artemisia scoparia*, and *Heteropappus altaicus*. Then the desert steppe has the lowest plant biodiversity that is dominated by *Stipa breviflora*, *Stipa bungeana*, *Peganum harmala*, *Salsola collina*, and *Lespedeza davurica*.

### ANPP measurement and regional estimation

A simple conventional method based on empirical relationship between *in situ* measurement of ANPP and the corresponding remotely sensed vegetation index (i.e., Normalized Difference Vegetation Index, NDVI) was used to estimate regional multi-year ANPP in the study area [30]. The *in situ* measurement of ANPP was taken as the peak aboveground biomass (including live biomass and standing dead biomass) during the growing season. This method is widely used to estimate grassland ANPP [31,32]. Vegetation surveys were conducted during peak growing seasons in 2011–2013 when aboveground biomass was maximum (i.e., in August). The sampling sites were randomly selected at intervals of c. 10–50 km across the entire Loess Plateau region to ensure representativeness. The sampling plots with natural re-vegetation were protected from human disturbances in order to prevent soil erosion and further land degradation. In particular, all sampling sites were protected from grazing herbivores. At each sampling site, aboveground biomass was clipped in three to five randomized 1 m × 1 m quadrats. The sampled biomass was dried at 65°C in an oven for 72 h and the dry weight then estimated. For the sites with shrub species, the twigs biomass for the current year was not measured. Thus shrub steppe ANPP was not estimated for the respective regions. ANPP data were collected from 188 sites, covering the three grassland types in the study area (Fig 1). The range of variation in ANPP was 25.4–569.8 g m^-2^ yr^-1^. Based on statistical analysis, the standard deviations of ANPP for the replicates at 82% of the sites were less than 20% of the mean value. This suggested that heterogeneity at each site was negligible in analysis of spatial patterns of ANPP at regional scale [10,17].

The peak monthly NDVI in 2011–2013 (in August) was used to determine the relation between *in situ* measurement of ANPP and the corresponding NDVI. Then the peak monthly NDVI in 2000–2013 (also in August) relative to the established ANPP-NDVI relation was used to estimate ANPP for each pixel of the study area. The NDVI data were derived from the Moderate Resolution Imaging Spectroradiometer (MODIS; https://lpdaac.usgs.gov). The peak monthly NDVI data for 2011–2013 (with 250 m × 250 m resolution) were obtained from MOD09GQ products, which provided daily surface-reflectance and NDVI datasets. The composite 1-day NDVI for August (corresponding to field sampling time) was averaged to get the monthly NDVI for August. The peak monthly NDVI data for 2000–2013 were from MOD13Q1 products (with the same spatial resolution of 250 m × 250 m) provided the 16-day composite NDVI.

In this study, the 1 km × 1 km NDVI were extracted from MOD13Q1 products with 4 raster cells for the whole study area. This was used to explore the spatial patterns of ANPP to match with corresponding climatic and LAI data for further analysis. The two 16-day NDVI composites for August were averaged to represent monthly NDVI for August. A variety of processing techniques (e.g., orbit calibration, sensor calibration, cloud screening, geo-referencing correction and atmospheric correction) were used to reduce the effects of cloud contamination, atmospheric perturbations and variable illumination and viewing geometry on the data products. A significant exponential correlation was noted between *in situ* measurement of ANPP and the corresponding NDVI for August—ANPP = 27.48e^3.70NDVI^ (*R* ^2^ = 0.71, *n* = 188, *P* < 0.001). The spatial pattern of ANPP for the entire region for 2000–2013 was thus estimated using this relationship and NDVI data for August. The regional ANPP data were thus used to explain the spatial variations in PUE.

### Precipitation interpolation

MAP data for 2000–2013 were from the China Meteorological Data Sharing Service System at http://cdc.cma.gov.cn/. This dataset contains climate data from 73 stations, 64 of which were selected based on the European Climate Assessment standards. The kriging method was used to interpolate the station-specific data at 1 km × 1 km resolution to create continuous data surfaces for meteorological factors. Then the data surface was re-sampled in ArcInfo GIS (v. 9.2) to derive specific meteorological factor for each sampling site and pixel.

Precipitation seasonal distribution (PSD) in the growing season (May to September) was quantified as coefficient of variation of the monthly precipitation (CV_mp_) as follows [10]:

$$\;CV_{\text{mp}}=\frac{\sqrt{\frac{1}{5}\sum_{i=5}^{9}{\left(M_{i}-\bar{M}\right)}^{2}}}{\bar{M}}$$ (1)

$$\;\bar{M}=\frac{1}{5}\sum_{\text{i}=5}^{9}\left(M_{i}\right)$$ (2)

where M_i_ is the averaged precipitation for month *i* (*i* from May to September); and $\;\bar{M}$ is the mean precipitation for the 5 months (May to September).

### Soil, topography and vegetation measurement properties

From the 188 sampling sites, 101 were long-term observation sites for monitoring changes in deep soil water content and ANPP. Soil samples at the 101 sites were collected after clipping aboveground biomass. At each sampling site, a 40 cm deep pit was excavated and undisturbed soil samples taken at three layer depths (0–10 cm, 10–20 cm and 20–40 cm) to measure saturated soil hydraulic conductivity (Ks, cm min^-1^) and soil bulk density (BD, g cm^-3^). Disturbed soil samples were also collected for laboratory analysis. The disturbed soil samples were air-dried and passed through 1 mm sieve and then analyzed for soil particle sizes using laser diffraction Mastersizer2000 (Malvern Instruments, Malvern, England). Volumetric soil water contents were simultaneously measured at the 101 sites using neutron probes placed a 20 cm interval to the depth of 500 cm. This gave the measure of the effect of soil water storage on ANPP and PUE. The slope gradient (°) and aspect (°) at each of the 101 sites was measured using geological compass. LAI data were from MODIS LAI product (1 km × 1 km; http://lpdaac.usgs.gov). Mean LAI was calculated for the peak growing season (August) in 2000–2013 as the maximum mean LAI for each pixel, corresponding to NDVI data.

### Data analysis

To determine the effects of MAP, PSD, vegetation and soil properties on spatial variations in ANPP and PUE in the entire Loess Plateau region, the 400, 1200 and 3000 sites were randomly selected respectively for meadow steppe, desert steppe and typical steppe based on the area of each grassland type. Prior to the site sampling, land use and land cover data (1 km × 1 km) for the Loess Plateau were used to eliminate non-grassland pixels. One-way ANOVA was used to determine the differences in ANPP and PUE for the three grassland types. For sites with significant differences, the least significant difference (LSD) test was conducted at *P* < 0.05. Regression analysis was used to test the associations among ANPP, PUE, vegetation, and soil properties. All statistical analyses were performed in SAS software (v. 8.0) (SAS Institute Inc., Cary, NC, USA).

## Results

### Spatial variations in ANPP and PUE

ANPP and PUE generally decreased with increasing aridity (*P* < 0.01) across the Loess Plateau. ANPP for the three grassland types varied significantly, in the range of 33.8–656.2 g m^-2^ yr^-1^ (Table 1). Meadow steppe had the highest average ANPP (380.2 g m^-2^ yr^-1^) and the lowest coefficient of variation (CV; 26.3%). Then typical steppe had medium ANPP (153.0 g m^-2^ yr^-1^) and the highest CV (60.0%). The lowest ANPP was for desert steppe (80.0 g m^-2^ yr^-1^) and with CV of 39.7%. Consistent with ANPP, there were significant differences in PUE among the three grassland types (Table 1). Meadow steppe had the highest PUE (0.92 g m^-2^ mm^-1^), followed by typical steppe (0.41 g m^-2^ mm^-1^) and then desert steppe (0.28 g m^-2^ mm^-1^).

**Table 1:** Statistical values of aboveground net primary productivity (ANPP) and precipitation-use efficiency (PUE) for the three grassland types: MS, meadow steppe (*n* = 400); TS, typical steppe (*n* = 3000); DS, desert steppe (*n* = 1200). CV% is the percent coefficient of variation: (Standard deviation/Mean). MAP_avg_ is the average mean annual precipitation (MAP) for the grassland types. Values followed by different lower-case letters are significantly different for different grassland types at *P* < 0.05.

	ANPP (g m^-2^ yr^-1^)			PUE (g m^-2^ mm^-1^)		
Variable	MS	TS	DS	MS	TS	DS
Minimum	54.8	43.0	33.8	0.17	0.10	0.11
Maximum	656.2	617.7	394.4	1.69	1.77	0.88
Mean	380.2a	153.0b	80.0c	0.92a	0.41b	0.28c
CV%	26.3	60.0	39.7	24.3	47.2	31.5
MAP_avg_	415.0	372.0	280.0	415.0	372.0	280.0

### Driving factors of ANPP and PUE

**Fig 2:**
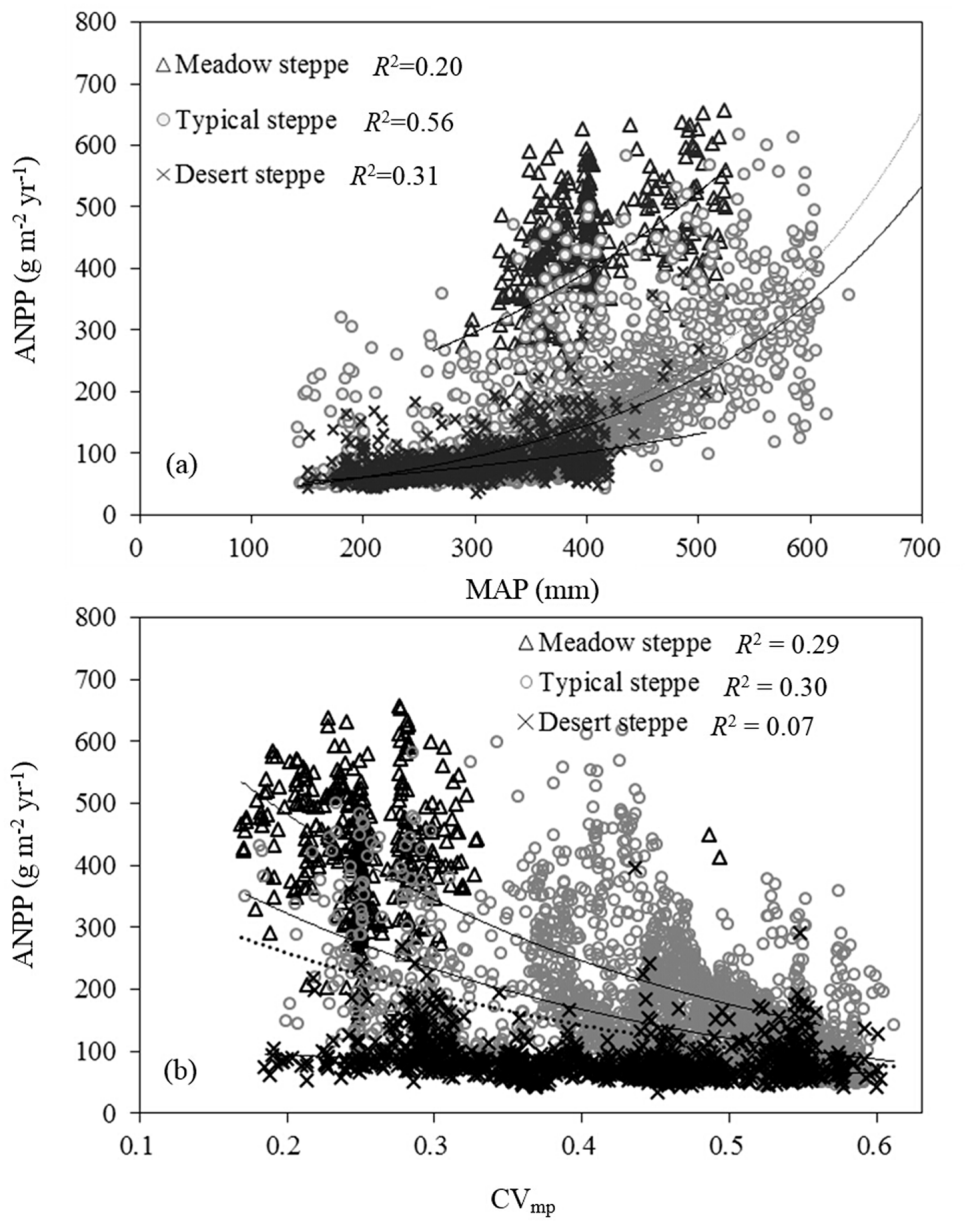
Correlation between (a) aboveground net primary productivity (ANPP) and mean annual precipitation (MAP) and that between (b) ANPP and precipitation seasonal distribution [PSD, quantified herein with coefficient of variation (CV_mp_)] and for each grassland type in the Loess Plateau temperate grassland. Each data point in the figure represents 14-year average value for 2000–2013. *R* ^2^ is the coefficient of determinant of the regression functions between MAP, CV_mp_ and ANPP for each grassland type.

Regression analysis was used to determine the effects of climatic, biotic and soil factors on the spatial variations in ANPP and PUE. ANPP for the entire temperate grassland in the Loess Plateau increased exponentially with increasing MAP (ANPP = 22.63e^0.048MAP^, *n* = 4600; *R* ^2^ = 0.50, *P* < 0.001), implying that an increase in PUE increased MAP (Fig 2a). The correlation was also exponential for typical steppe and desert steppe. However, a linear MAP-ANPP correlation (*R* ^2^ = 0.21, *P* < 0.001) was observed for meadow steppe. MAP accounted for 56% (by *R* ^2^ analysis) of the spatial variations in ANPP in typical steppe, 31% in desert steppe and 21% in meadow steppe.

Inconsistent with MAP, there was significant negative correlation between ANPP and CV_mp_ (*R* ^2^ = 0.29, *P* < 0.001), suggesting that PSD had a strong impact on spatial variations in ANPP at regional scale (Fig 2b). Although negative correlations were also noted for all the grassland types, *R* ^2^ values were different (*P* < 0.001). Regression analysis showed that CV_mp_ accounted for 29% and 30% of the spatial variations in ANPP respectively in meadow steppe and typical steppe. However, CV_mp_ only explained 7% of the spatial variations in ANPP in desert steppe.

Using *in situ* measurement of ANPP and soil moisture for the 101 sites, the effects of SWS at various soil layer depths on ANPP were determined (Fig 4a). Consistent with MAP, ANPP was positively correlated with SWS for the various soil layer depths and it increased exponentially with increasing SWS (*P* < 0.001). However, the ability of SWS to explain the variations in ANPP decreased with increasing soil depth. For example, the 0–1 m layer SWS accounted for 29% of the spatial variations in ANPP and the 4–5 m layer SWS for only 9%. Note that MAP explained more spatial variations in ANPP (49%) than SWS (Fig 4a). This implied that regional patterns of ANPP were controlled more by precipitation than by soil moisture in the Loess Plateau.

**Fig 3:**
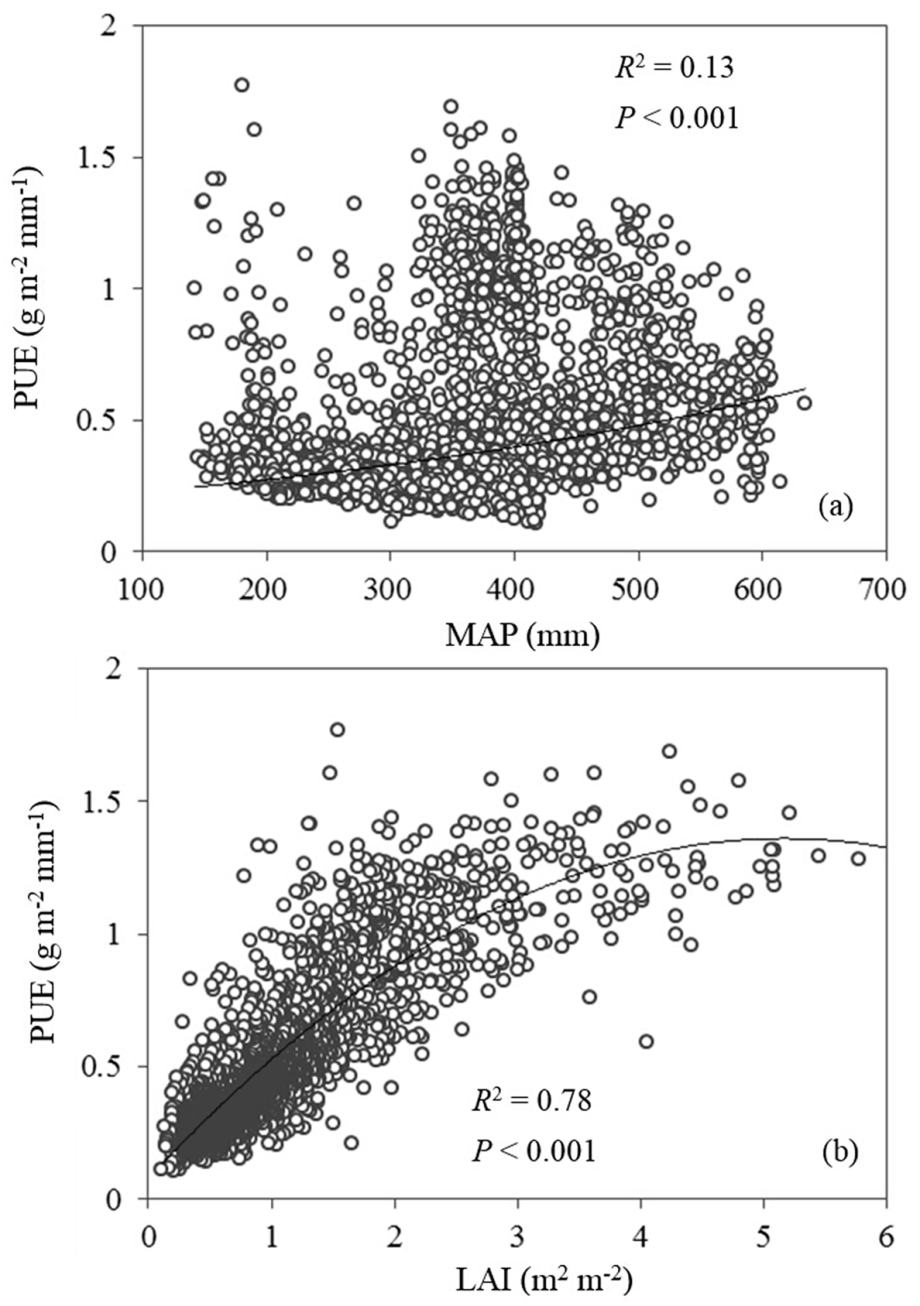
Correlations between (a) precipitation use efficiency (PUE) and mean annual precipitation (MAP) and that between (b) PUE and leaf area index (LAI) for the entire Loess Plateau temperate grasslands.

**Fig 4:**
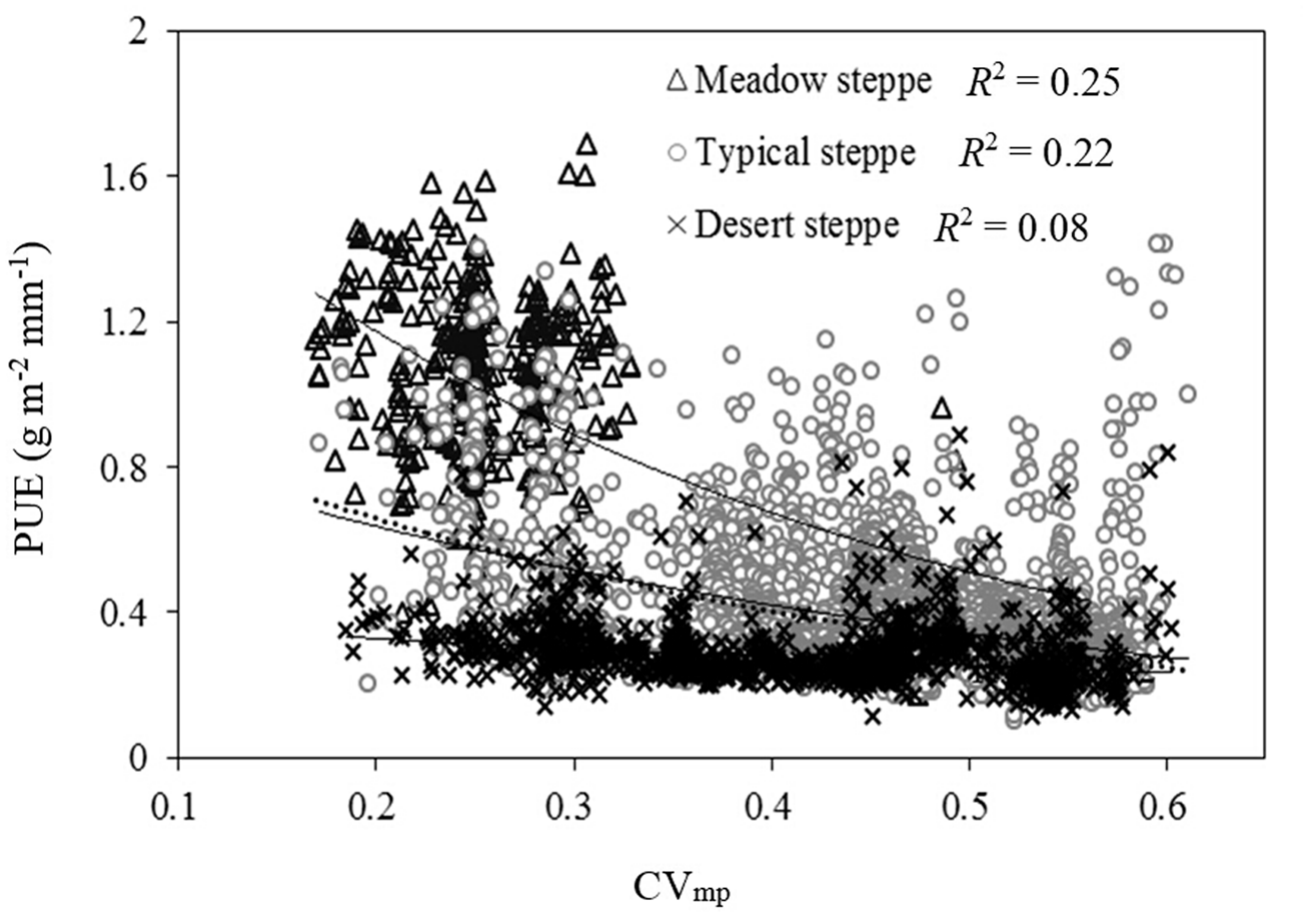
Correlation between precipitation use efficiency (PUE) and precipitation seasonal distribution (PSD, quantified herein with CV_mp_) for each grassland type and for the entire Loess Plateau temperate grassland.

**Fig 5:**
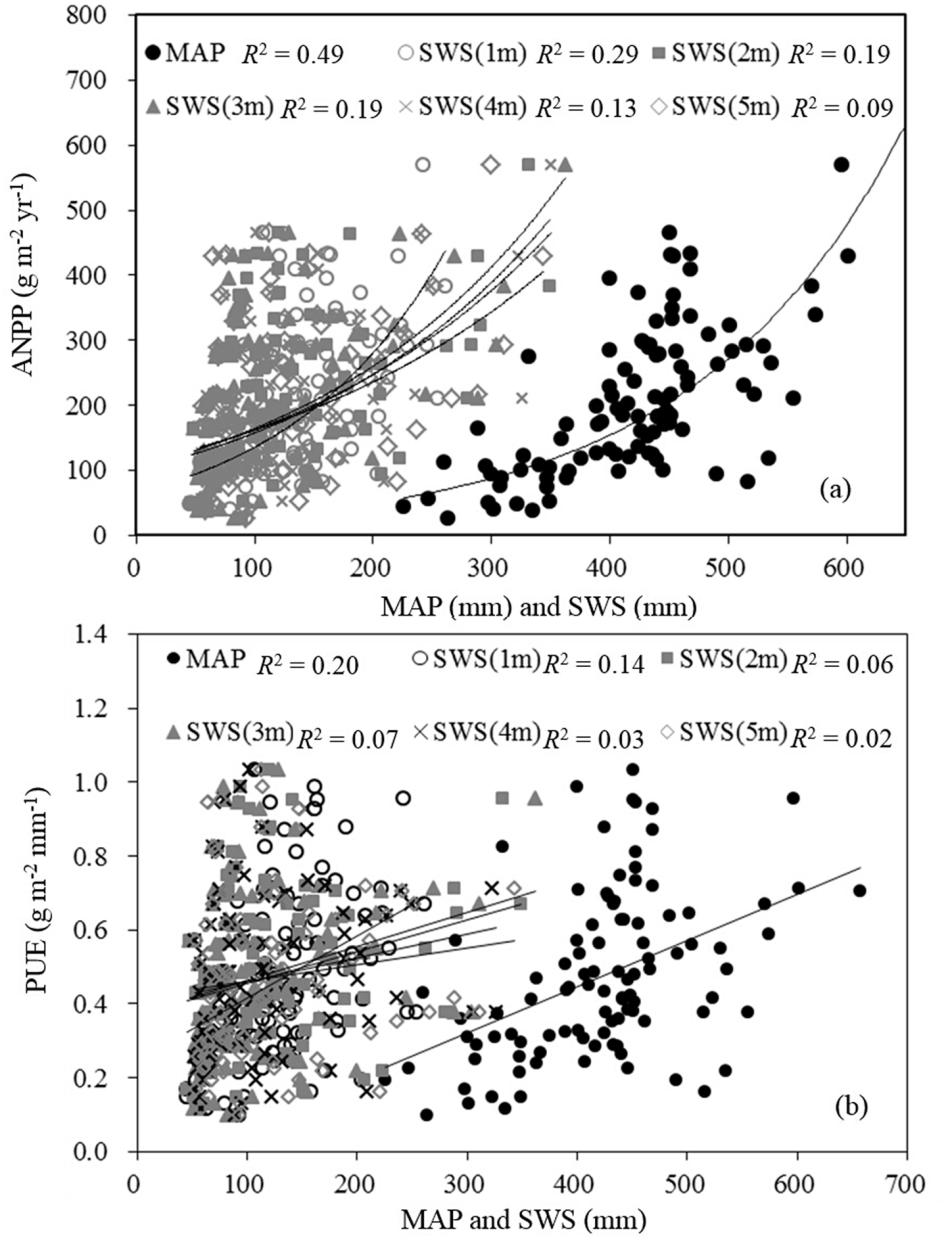
Correlation between (a) aboveground net primary productivity (ANPP) and mean annual precipitation (MAP) and that separately between (b) precipitation-use efficiency (PUE) and MAP and also soil water storage (SWS) at various soil depths at the 101 soil water observation sites in the Loess Plateau grassland.

Consistent with ANPP, PUE was positively correlated with MAP (*R* ^2^ = 0.13, *P* < 0.001) and negatively correlated with PSD (*R* ^2^ = 0.29, *P* < 0.001) for the Loess Plateau temperate grassland (Fig 5a and Fig 3). Besides, a positive correlation existed between PUE and LAI. LAI explained 78% of the variations in PUE in temperate Loess Plateau grasslands (Fig 5b). SWS for various soil depths also had a positive effect on PUE, but the ability to explain PUE decreased with increasing soil depth (Fig 4b). The above results suggested that the ability of climatic and soil factors to explain spatial variations in ANPP was much weaker than that of biotic factors. In contrast to the above results, soil texture and other soil physical properties (e.g., Ks, BD) were not significantly correlated with ANPP and PUE (data not shown).

Furthermore, the effects of slope gradient and aspect on ANPP and PUE were determined using *in situ* measurement of ANPP, slope gradient and aspect at the 101 sites. The analysis showed that ANPP was negatively correlated with slope gradient (*P* = 0.03, *n* = 101). PUE was also negatively correlated with slope gradient, although not statistically significant (*P* = 0.063, *n* = 101). No significant correlation was noted between aspect and ANPP or PUE (data not shown).

## Discussions

### Characteristics of ANPP and PUE

Compared with both typical steppe and desert steppe, meadow steppe had the highest ANPP and PUE in the Loess Plateau. This trend could be attributed to favorable environmental conditions in the region. Similarly, previous studies noted that drier sites tend to have lower PUE because of low plant density, low production potential, high evaporation potential and high tolerance to water stress [1,13,15,17]. Meadow steppe is mainly in the west of the Loess Plateau where water and heat conditions (driven by southeast monsoon winds) favor plant growth [33]. Other studies also noted that higher precipitation, lower temperature and thus lower potential evapotranspiration rates favor higher ANPP and PUE in meadow steppes [10,17]. PUE for temperate grasslands in the Loess Plateau (0.28–0.94 g m^-2^ mm^-1^) is similar to that (0.05–1.81 g m^-2^ mm^-1^) reported by Le Houérou et al. [34] for grassland ecosystems in arid and semiarid regions in the world. Furthermore, mean PUE for temperate grasslands in the Loess Plateau is also close to those reported by Bai et al. [15] and Hu et al. [17]. PUE for meadow steppe was significantly different from that for typical steppe under similar precipitation condition (Table 1). This could be because typical steppe is in areas with the highest water and soil erosion in the Loess Plateau [35], resulting in severe precipitation loss by runoff and soil water evaporation. This reduces the ratio of transpiration to annual precipitation, which in turn reduces PUE [17]. Furthermore, severe soil and water erosion could cause loss of nutrients, renders soils infertile and thus lower ANPP. It was therefore conclude that PUE of grassland ecosystems depended on land surface patterns such as topography and landforms.

### Factors affecting variations in ANPP

Precipitation, a proxy for water availability, is a major determinant of vegetation production at regional and global scales, especially in arid and semiarid regions [1,10,13,36]. This study showed an exponential positive relation between ANPP and MAP for the Loess Plateau temperate grasslands. This suggested that increase in MAP causes increase in PUE, which supported the first hypothesis in this study. This result is also consistent with that reported in most previous studies [10,12,17,37]. Studies on grassland ecosystems in other regions of the world reported linearity as the most common relation between ANPP and MAP [6,12,13]. Hu et al. [17] concluded that inconsistencies could be due to insufficient sampling sites in arid regions, which conclusion was also supported by Guo et al. [10]. The findings of this study also confirmed this point. Using data from a large randomly-selected sample sites, a linear MAP-ANPP relation was observed for meadow steppe. However, the function was exponential for the sites in the three grassland types. This was due to possible differences in plant functional characteristics such as sensitivity to changes in rainfall [7,10,13,14]. Obviously, the explanatory exponential nature of MAP for variations in ANPP of desert steppe (31%) and meadow steppe (20%) was substantially weaker than that for the whole grassland types (50%) and/or typical steppe (56%). This trend could be related to the range of variation in MAP. MAP range was 265–520 mm for meadow steppe and 150–500 mm for desert steppe. However, typical steppe had the widest range of variation in MAP (140–640 mm). Note that range included that of MAP for the entire temperate grassland in the Loess Plateau. The results suggested that the effects of MAP on ANPP somehow depended on the spatial scale used. The results of this study are in agreement with those reported in other studies. Several other studies have observed significant correlation between ANPP and MAP at regional scale, but relatively weak correlation at site scale [10,17,24].

Like MAP, PSD had a significant effect on spatial variations in ANPP in the Loess Plateau temperate grassland ecosystems. The results showed that more even PSD favored higher ANPP in the Loess Plateau, supporting the second hypothesis of this study. However, this finding was inconsistent with others where more concentrated PSD improved ANPP [10,18,20]. The differences in the ANPP patterns could be due to the differences in the characteristics of the climate, topography, landforms, soil and parent materials. As loess is subject to high erosion, a unique morphology has developed in the Loess Plateau. The typical landforms in the region are Loess “Yuan, Liang and Mao” and valleys of different magnitudes of erosion. Hills, deep gullies and undulating loess slopes are very common and are the characteristic landscapes of the Plateau region [35]. Subsequently, infrequent, but more extreme rainfall events, without concurrent changes in total precipitation could intensify soil and water erosion in the region [27,28,35]. This increases water and nutrient loss via runoff, lowers precipitation water infiltration into deep soil layers and reduces soil water content during growing season, thereby limiting plants photosynthesis.

The negative correlations between slope gradient and measured ANPP for the 101 observation sites could explain the issue of water and nutrient loss via runoff. The spatial variations in ANPP could be related with the complexity of the terrain. Other studies noted that concentrated PSD could enhance precipitation water infiltration into deep soil layers and thereby lower water loss via evaporation [10,18,19,20]. However, weak evaporation water loss under concentrated PSD could not compensate for enhanced precipitation water loss via runoff because of complex terrain, sparse vegetation and loose soil in the Loess Plateau. Longer dry spells between rainfall events due to enhanced CV_mp_ plus excessively waste of concentrated precipitation water via overland flow could extend the period of below-average soil water content. Decrease in soil water availability could increase plant water stress, and thereby force plants to maintain low rates of photosynthesis. This is especially holds for low-stress tolerance plants, a condition that could limit ANPP [19]. Also intensified soil and water erosion due to concentrated PSD could cause loss of nutrients and plant seeds in slope lands, thereby reducing plant species diversity and thus ANPP [15,32].

This study showed that MAP contribution to spatial variations in ANPP was greater than that of PSD for the Loess Plateau temperate grassland. This suggested that changes in PSD due to climate change are not commensurate to changes in total annual precipitation. For the entire temperate grassland, spatial variations in ANPP for typical and desert steppes were more affected by MAP than by PSD. However, the reverse trend was noted for meadow steppe. Thus as noted by Guo et al. [10], the relative significance of MAP and PSD depended on grassland type. Guo et al. [10] attributed this difference to the different compositions of functional plant types in different grassland types.

In addition to MAP and PSD, the effects of SWS at various soil depths on spatial variations in ANPP were determined using *in situ* measurement of ANPP. Consistent with MAP, ANPP increased exponentially with increasing SWS. However, the effect of SWS on the spatial variations in ANPP was less than that of MAP. Furthermore, the ability of SWS to explain the variations in ANPP decreased with increasing soil depth. This could be related with the distribution of plant roots in the soil. With the exception of some artificial grassland (e.g., alfalfa), roots in the temperate Loess Plateau grasslands are predominantly distributed in the 0–40 cm soil layer [38,39]. Furthermore, root index (length density, root weight, root surface area and root diameter) generally decrease with increasing soil depth. The maximum root index is for the 0–30 cm soil layer [39,40]. Decreasing root index with increasing soil depth reduces deep soil water uptake by plants, and thereby weakens plant-soil water relation. Thus MAP was the dominant factor driving spatial variations in ANPP for the entire temperate Loess Plateau grasslands. Also the effect of PSD on the spatial variations in ANPP of a given grassland type cannot be ignored, especially for meadow steppes.

### Factors affecting variations in PUE

MAP, PSD, LAI and SWS had significant effects on the spatial variations in PUE in Loess Plateau temperate grassland. The results of this study were in good agreement with the results of several other studies [15,17,23,41] where PUE increased with increasing MAP. McNaughton [42] showed that PUE increased within 0.17–0.94 g m^-2^ mm^-1^ along a precipitation gradient of 480–1150 mm yr^-1^ in the Serengeti grassland of East Africa. Paruelo et al. [13] investigated spatial variations in PUE across a precipitation gradient of 200–1200 mm yr^-1^ and noted that PUE increased initially before decreasing with increasing MAP, peaking at MAP = 475 mm. The results here agreed with this pattern of annual precipitation range of 280–415 mm across the three grassland types (Table 1). However, other reports observed constant spatial PUE [2,6,24,43], while Huxman et al. [7] noted negative correlation between PUE and MAP across North America. Hu et al. [17] observed that PUE increased initially and then decrease with increasing MAP at continental and global scales. These discrepancies could be ascribed to the use of different MAP ranges and ecosystem types. Irrespectively, PUE for temperate Loess Plateau grasslands increased with increasing MAP. This is because in water-limited regions, vegetation canopies grow close together and plants have much higher growth rates with increasing MAP. Thus plants use more precipitation for productivity and for transpiration.

Contrary to MAP, PUE decreased with increasing CV_mp_. This implied that concentrated PSD weakened PUE of the temperate Loess Plateau grasslands. This is because there was more precipitation lost (via runoff) with increasing rainfall variability. Also severe soil and water erosion [35] meant less available precipitation water for plant use. These conditions reduced ANPP and thus PUE. The contribution of PSD to the variations in PUE was higher under meadow steppe and typical steppe than under desert steppe (Fig 5). This also suggested that ecosystem carbon and water cycle was less sensitive to changes in PSD in drier environments. This could be ascribed to the relatively low PUE and high water stress tolerance at direr sites. Compared with MAP, PSD explained more variations in PUE for the entire temperate Loess Plateau grassland. Thus changes in PSD due to climate change could have more effect on PUE than changes in total annual precipitation. This aspect hydrology is largely understudied.

Except for climatic factors, there was a significant correlation between LAI and PUE in the Loess Plateau. LAI explained 78% of the spatial variations in PUE, much higher than that of MAP and PSD. This is consistent with the findings of Hu et al. [22] where LAI was reported as the primary driving factor of spatiotemporal variations in water use efficiency. LAI significantly influences carbon assimilation and transpiration to evapotranspiration ratio [44,45]. Furthermore, regions with relatively high LAI exhibit low runoff; which element conserves soil water for plant use [14] and thus causes high PUE. This study showed the feasibility of predicting PUE from LAI in grassland ecosystems. No significant correlation was noted between soil properties and PUE, except for SWS. Consistent with ANPP, the effects of SWS on PUE also decreased with increasing soil depth. This was ascribed to the pattern of distribution of plant roots along soil depth. It was therefore concluded that changes in both vegetation structure (and thus in LAI) and PSD could significantly influence ecosystem carbon and water cycle in grasslands. Furthermore, several studies have shown different spatial and temporal variations in PUE. This underscores the underlying differences in regional and local ecological processes [15,17]. Thus there is the need for further studies on the temporal patterns of PUE and the controlling factors, especially in China’s Loess Plateau region.

## Conclusions

In this study, we quantified the effects of climatic, biotic and soil factors on spatial variations in ANPP and PUE in Loess Plateau temperate grassland. We noted exponential correlation between MAP and ANPP for the Loess Plateau temperate grassland, suggested that PUE increased with increasing MAP. Our study suggested that LAI was the primary factor controlling the spatial variations in PUE in the entire Loess Plateau temperate grassland. This showed the feasibility for easily predicting PUE from LAI in grassland ecosystems. Both ANPP and PUE decreased with increasing CV_mp_, suggesting that more even precipitation distribution favored higher ANPP and PUE in the Loess Plateau grasslands. We therefore concluded that changes in vegetation structure, LAI and in the patterns of seasonal distribution of rainfall due to global climate change could significantly influence ecosystem carbon and water cycle in grasslands. Further research was needed to determine temporal response of ANPP and PUE to variations in climatic and biotic factors in the Loess Plateau grasslands.
